# FEASIBILITY OF A LIFESTYLE-INTEGRATED EXERCISE DELIVERED VIA AN MHEALTH PLATFORM IN PREFRAIL OLDER ADULTS

**DOI:** 10.1093/geroni/igae098.2760

**Published:** 2024-12-31

**Authors:** Na Li, Nan Wang, Siyang Lin, Yin Yuan, Feng Huang, Pengli Zhu

**Affiliations:** Fujian Provincial Hospital, Fuzhou, Fujian, China (People’s Republic); Fujian Medical University, Fuzhou, Fujian, China (People’s Republic); Shengli Clinical Medical College of Fujian Medical University, Fuzhou, Fujian, China (People’s Republic); Fujian Provincial Hospital, Fuzhou, Fujian, China (People’s Republic); Fujian Provincial Hospital, Fuzhou, Fujian, China (People’s Republic); The Shengli Clinical Medical College of Fujian Medical University, Fuzhou, Fujian, China (People’s Republic)

## Abstract

**Background:**

Lifestyle-integrated Functional Exercise, which integrates exercise into daily life, may be a promising alternative to overcome traditional exercise barriers. Recent advances in mHealth technology hold the promise of preserving the benefits of supervised exercise training in group settings by remotely guiding and tracking exercise progress. Objective: To assess the feasibility and acceptability of an mHealth platform to deliver a personalized lifestyle-integrated multicomponent exercise program for pre-frail individuals.

**Methods:**

This was a community-based, mixed-methods, prospective, single-cohort feasibility study. All participants were prescribed a lifestyle-integrated multicomponent exercise program by healthcare professionals using the PF-Life platform (web-based portal), and participants engaged in customized exercise following in-app video instructions and feedback on the PF-Life platform (smartphone application). Physical activity was registered daily through wearable devices. Result：16 participants successfully completed the study with a retention rate of 100%. Mean adherence to the number of days exercised per week was 88.8% (95% CI 75%-100%). Changes were observed from baseline to follow-up for total Handgrip strength (21.41±6.38 vs. 24.12± 6.62 kg, d =0.714), TUG (8.23±1.33 vs. 7.48± 2.01 seconds, d=1.647) and 30-second chair rise test (17.13±4.3 vs. 20.04±4.54 repetitions, d =0.94). The sedentary time decreased by a mean of 33 minutes/day and low physical activity increased by 31 minutes/day during the intervention.

**Conclusion:**

This study provides preliminary evidence that an lifestyle-integrated exercise based on mHealth platform has good adherence, safety, reliability and usability, with initial results in reducing sedentary time and improving physical function.

